# *Bacteroides thetaiotaomicron* metabolic activity decreases with polysaccharide molecular weight

**DOI:** 10.1128/mbio.02599-23

**Published:** 2024-02-20

**Authors:** Jeremy P. H. Wong, Noémie Chillier, Michaela Fischer-Stettler, Samuel C. Zeeman, Tom J. Battin, Alexandre Persat

**Affiliations:** 1 Institute of Bioengineering and Global Health Institute, School of Life Sciences, École Polytechnique Fédérale de Lausanne, Lausanne, Switzerland; 2 School of Architecture, Civil and Environmental Engineering, École Polytechnique Fédérale de Lausanne, Sion, Switzerland; 3 Department of Biology, ETH Zürich, Zürich, Switzerland; University of Pittsburgh School of Medicine, USA

**Keywords:** *Bacteroides*, PULs, polysaccharides, microbial communities, cross-feeding, gut microbiota

## Abstract

The human colon hosts hundreds of commensal bacterial species, many of which ferment complex dietary carbohydrates. To transform these fibers into metabolically accessible compounds, microbes often express a series of dedicated enzymes homologous to the starch utilization system (Sus) encoded in polysaccharide utilization loci (PULs). The genome of *Bacteroides thetaiotaomicron* (*Bt*), a common member of the human gut microbiota, encodes nearly 100 PULs, conferring a strong metabolic versatility. While the structures and functions of individual enzymes within the PULs have been investigated, little is known about how polysaccharide complexity impacts the function of Sus-like systems. We here show that the activity of Sus-like systems depends on polysaccharide size, ultimately impacting bacterial growth. We demonstrate the effect of size-dependent metabolism in the context of dextran metabolism driven by the specific utilization system PUL48. We find that as the molecular weight of dextran increases, *Bt* growth rate decreases and lag time increases. At the enzymatic level, the dextranase BT3087, a glycoside hydrolase (GH) belonging to the GH family 66, is the main GH for dextran utilization, and BT3087 and BT3088 contribute to *Bt* dextran metabolism in a size-dependent manner. Finally, we show that the polysaccharide size-dependent metabolism of *Bt* impacts its metabolic output in a way that modulates the composition of a producer-consumer community it forms with *Bacteroides fragilis*. Altogether, our results expose an overlooked aspect of *Bt* metabolism that can impact the composition and diversity of microbiota.

**IMPORTANCE:**

Polysaccharides are complex molecules that are commonly found in our diet. While humans lack the ability to degrade many polysaccharides, their intestinal microbiota contain bacterial commensals that are versatile polysaccharide utilizers. The gut commensal *Bacteroides thetaiotaomicron* dedicates roughly 20% of their genomes to the expression of polysaccharide utilization loci for the broad range utilization of polysaccharides. Although it is known that different polysaccharide utilization loci are dedicated to the degradation of specific polysaccharides with unique glycosidic linkages and monosaccharide compositions, it is often overlooked that specific polysaccharides may also exist in various molecular weights. These different physical attributes may impact their processability by starch utilization system-like systems, leading to differing growth rates and nutrient-sharing properties at the community level. Therefore, understanding how molecular weight impacts utilization by gut microbe may lead to the potential design of novel precision prebiotics.

## INTRODUCTION

Polysaccharides are complex, polymeric molecules composed of long chains of monosaccharide subunits linked together through glycosidic linkages (1). These molecules are essential to life (2) and are commonly found in our daily diets (3). Their chemical structures are variable due to differences in monosaccharide compositions and glycosidic linkage patterns (4). As a result, the degradation of these molecules requires diverse sets of enzymes that generally target specific polysaccharides. While humans lack the ability to degrade many polysaccharides, their intestinal microbiota contain bacterial commensals that are versatile polysaccharide utilizers. In fact, microbiota species are selected for the ability to forage more or less complex carbohydrates and glycans (5). Thus, for many gut microbes, the ability to persist in the gut correlates with their ability to utilize dietary fibers and other carbohydrate-based nutrients (6). Therefore, understanding the mechanisms of polysaccharide utilization by gut microbes may provide an understanding to the forces driving the composition of the gut microbiota (5).

*Bacteroides* spp. are among the most abundant and well-studied glycan-foraging commensals found in the human bowel (7). *Bacteroides* can utilize complex carbohydrates through the expression of a series of protein systems encoded in polysaccharide utilization loci (PULs) (8). PULs encode for proteins that bind, cleave, and import complex polysaccharides into simpler, metabolically accessible sugars (9–13). *Bacteroides thetaiotaomicron* (*Bt*) can utilize many dietary polysaccharides including starch (14), arabinan (15), levan (16), inulin (17), rhamnogalacturonan II (18), complex dietary N-glycans (19), and even glycans derived from its mammalian hosts such as o-linked glycans from mucins and human milk oligosaccharides (20).

The starch utilization system (Sus) has served as a model system for PUL studies (14). In *Bt*, eight genes encode Sus (*susRABCDEFG*). SusD, SusE, or surface glycan-binding protein (SGBP) and SusF are outer membrane lipoproteins that are responsible for the initial binding of oligosaccharides to the cell surface (14, 21). SusG is an outer membrane-associated α-amylase that hydrolyzes the starch into maltooligosaccharides (22). The resulting maltooligosaccharides enter the periplasm thanks to the TonB-dependent transporter SusC (23). These sugars are further cleaved by two periplasmic glycoside hydrolases (GHs) SusA (neopullulanase) and SusB (α-glucosidase) (24, 25). The inner membrane-spanning sensor-regulator protein SusR regulates the expression of Sus genes in response to periplasmic levels of maltose (26). Other PULs are Sus homologs encoding the nutrient-binding and importer proteins SusD and SusC, lipoproteins of similar structures to SGBPs and SusF, along with a series of diverse GHs dedicated to a wide range of different complex carbohydrates, which are regulated by a SusR-like protein (27). The genome of *Bt* encodes 96 different PULs (28), allowing them to grow on a broad range of dietary or host-derived glycans (9, 10, 12, 19).

Although different PULs are dedicated to the degradation of specific polysaccharides with unique glycosidic linkages and monosaccharide compositions, a specific polysaccharide may be found with different complexities such as molecular weight (MW) and degree of branching (4), as in starch produced by different plants (29). Sus-like systems are composed of multiple surface-exposed components that need to coordinate functions to properly cleave and import degraded oligosaccharides. These must, therefore, handle polymers with the vastly differing physical attributes that may impact their processability. However, little is known about how MW impacts the efficacy of PULs in producing accessible glycans that act as accessible substrate for growth.

In a previous work (30), we have identified dextran, a polysaccharide commonly used as an additive in the food industry (31), which is readily utilized by *Bt*. Like starch, dextran is a polyglucan consisting of two unique glycosidic linkages: an α-1,6-linked backbone along with α-1,3 branches (32). It is known that the degradation of dextran involves the enzyme dextranase, where they may be further classified into two separate classes: endodextranase or exodextranase (33). Endodextranases (GH families 31, 49, and 66) act by randomly hydrolyzing the α-(1→6) linkages of dextran, which leads to the formation of isomaltooligosaccharides of various degrees of polymerization, whereas exodextranases (GH families 13, 15, 27, and 49) act on the non-reducing end of dextran, forming glucose, isomaltose, or isomaltotriose as the main degradation products (33). Previous works have identified the upregulation of PUL48 in *Bt* under dextran growth (30, 34), which is structurally very similar to a PUL dedicated to the utilization of dextran in the Gram-negative soil bacterium *Flavobacterium johnsoniae* (33). Both PULs featured a GH66 and a GH31, which likely serve as endodextranase and exodextranase for the hydrolysis of dextran, respectively. The GH family GH66 contains two main classes of enzymes: endodextranases and cycloisomaltooligosaccharide glucanotransferases, which hydrolyze dextrans via cyclization. It has been previously shown that *Bt*’s GH66, BT3087, possesses both endodextranase and cycloisomaltooligosaccharide glucanotransferase activity (35).

Unlike starch, dextran polymers are highly soluble in water. Also, due to their extensive commercial and clinical applications, there exist many purification protocols that produce dextran polymers of defined MW (31). Using *Bt* as the model commensal and dextran as the model dietary fiber, we investigated the impact of polysaccharide MW on the efficacy of dextran utilization by *Bt*. We found that *Bt* growth rate decreases as dextran MW increased. By studying dextran metabolism with *Bt* mutants containing knockouts of PUL48 genes, we identified protein targets within PUL48 whose importance in dextran utilization is MW-dependent, thereby suggesting a mechanism of size-dependent metabolism. We ultimately demonstrate that size-dependent metabolism has an impact on the composition of a model microbiota community.

## RESULTS

### *Bt* uses PUL48 to degrade dextran

**Fig 1:**
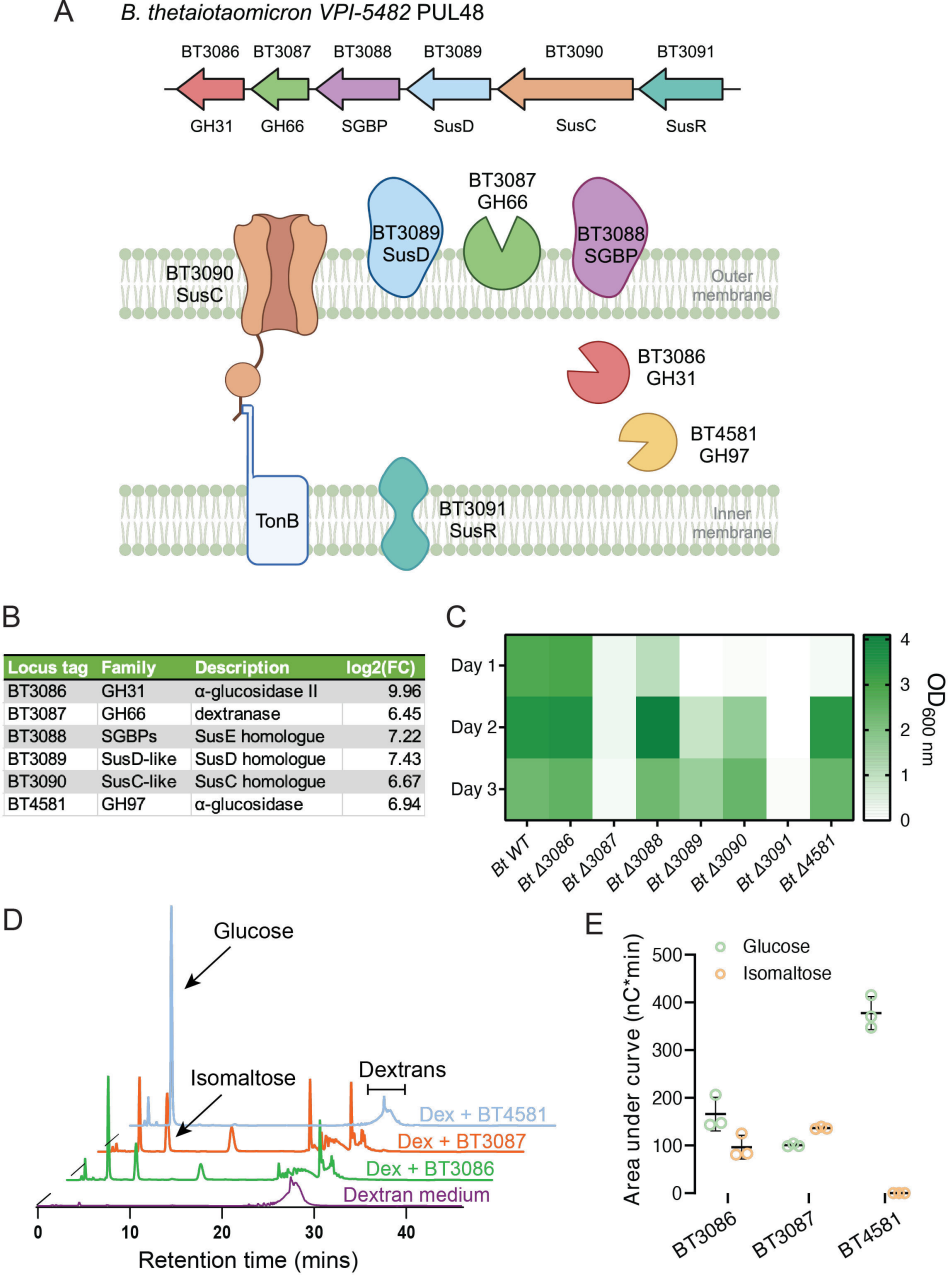
The roles of PUL48 proteins in the mechanism of dextran metabolism. (**A**) Genomic organization of *Bt’*s PUL48 along with a schematic of its structure based on previous works focused on other PULs of *Bt* or other similar organisms (5, 15, 17, 19, 37–39). (**B**) List of proteins that are significantly upregulated in dextran along with their functions. These proteins include six proteins belonging to the predicted PUL48, along with a sole GH BT4581. Log_2_(FC) corresponds to the log ratio of a protein’s expression values in *Bt* grown in dextran versus in glucose. (**C**) Monoculture growths of *Bt* WT and various PUL48 KO mutants in dextran. We measured the OD of individual cultures everyday over the course of 3 days. (**D**) HPAEC-PAD analysis of dextran medium treated overnight with recombinantly expressed GHs: BT3086, BT3087, or BT4581. Violet, no treatment; green, treatment with BT3086; orange, treatment with BT3087; and blue, treatment with BT4581. (**E**) Area under the curve analysis of the glucose and isomaltose peaks of dextran treated with BT3086, BT3087, or BT4581. BT3086 and BT3087 form both glucose and isomaltose as products, whereas BT4581 forms solely glucose. HPAEC-PAD experiments were run in triplicates; we display one representative chromatogram.

*Bt* exposed to dextran as the sole carbon source upregulates proteins belonging to the predicted PUL48 and the α-glucosidase BT4581 (30) (Fig. 1A and B). Consistent with this, a functional genomic screen identified genes from PUL48 to be essential for growth in dextran (36). PUL48 comprises a SusD-like surface-associated nutrient-binding protein (BT3089), a SusC-like TonB-dependent importer protein (BT3090), a SusR-like sensor-regulator protein (BT3091), and two GHs: α-glucosidase II (BT3086) and dextranase (BT3087) (Fig. 1A and B). To study the functions of individual PUL48 proteins in *Bt* dextran metabolism, we generated knockout mutants of PUL48 genes (Table 1). We first characterized the essentiality of these genes for growth in dextran. We grew individual mutants in *Bacteroides* minimal medium (BMM) supplemented with dextran (MW = 35 kDa) over 3 days (Fig. 1C). We observed that *Bt* mutants lacking the α-glucosidase II BT3086 and nutrient-binding protein BT3088 (SGBP) resulted in marginal growth defects in dextran. Mutants lacking the α-glucosidase BT4581 showed delayed growth. Mutants in nutrient-binding protein gene BT3089 (SusD) and importer protein BT3090 (SusC) showed severely impaired growth in dextran in comparison to wild-type (WT) *Bt*. Finally, *Bt* mutants in the regulatory protein BT3091 (SusR) and the dextranase BT3087 were entirely unable to grow in dextran (Fig. 1C). We examined the growth profiles of *Bt* mutants lacking several GHs: *Δ86,81* (double deletion of BT3086 and BT4581), *Δ87,81* (double deletion of BT3087 and BT4581), *Δ86,87* (double deletion of BT3086 and BT3087), and *Δ86,87,81* (triple deletion of BT3086, BT3087, and BT4581) also suggest that cell growth is eliminated only in the case of the loss of dextranase BT3087 (Fig. S1A). These results suggest that the proteins BT3086, BT3088, and BT4581 are auxiliary proteins that promote but are not essential to dextran fermentation. The proteins BT3089 (SusD) and BT3090 (SusC) are important for the proper growth of *Bt* in dextran. Their absence likely leads to a decrease in nutrient availability to *Bt* through impaired oligosaccharide binding and import.

**TABLE 1:** List of strains and plasmids generated for this study

Reagent type	Background	Designation	Source	Description
Strain	*Bt VPI-5482*	*Bt* WT	Whitaker et al. (40)	*Bt* WT
Strain	*Bt VPI-5482*	*Bt* WT - sfGFP	Whitaker et al. (40)	*Bt* WT constitutively expressing sfGFP
Strain	*Bt VPI-5482*	*Bt* WT - mCherry	Whitaker et al. (40)	*Bt* WT constitutively expressing mCherry
Strain	*Bt VPI-5482*	*Bt ΔBT3086*	This study	Deletion of α-glucosidase II BT3086
Strain	*Bt VPI-5482*	*Bt ΔBT3087*	This study	Deletion of dextranase BT3087
Strain	*Bt VPI-5482*	*Bt ΔBT3088*	This study	Deletion of SGBP BT3088
Strain	*Bt VPI-5482*	*Bt ΔBT3089*	This study	Deletion of SusD-like BT3089
Strain	*Bt VPI-5482*	*Bt ΔBT3090*	This study	Deletion of SusC-like BT3089
Strain	*Bt VPI-5482*	*Bt ΔBT3091*	This study	Deletion of SusR-like BT3089
Strain	*Bt VPI-5482*	*Bt ΔBT4581*	This study	Deletion of α-glucosidase BT4581
Strain	*Bt VPI-5482*	*Bt ΔBT3086* sfGFP	This study	*Bt ΔBT3086* constitutively expressing sfGFP
Strain	*Bt VPI-5482*	*Bt ΔBT3087* sfGFP	This study	*Bt ΔBT3087* constitutively expressing sfGFP
Strain	*Bt VPI-5482*	*Bt ΔBT3088* sfGFP	This study	*Bt ΔBT3088* constitutively expressing sfGFP
Strain	*Bt VPI-5482*	*Bt ΔBT3089* sfGFP	This study	*Bt ΔBT3089* constitutively expressing sfGFP
Strain	*Bt VPI-5482*	*Bt ΔBT3090* sfGFP	This study	*Bt ΔBT3090* constitutively expressing sfGFP
Strain	*Bt VPI-5482*	*Bt ΔBT3091* sfGFP	This study	*Bt ΔBT3091* constitutively expressing sfGFP
Strain	*Bt VPI-5482*	*Bt ΔBT4581* sfGFP	This study	*Bt ΔBT4581* constitutively expressing sfGFP
Strain	*Bt VPI-5482*	*Bt ΔBT3088* mCherry	This study	*Bt ΔBT3088* constitutively expressing mCherry
Strain	*Bt VPI-5482*	*Bt ΔBT3089* mCherry	This study	*Bt ΔBT3089* constitutively expressing mCherry
Strain	*Bt VPI-5482*	*Bt ΔBT3090* mCherry	This study	*Bt ΔBT3090* constitutively expressing mCherry
Strain	*Bt VPI-5482*	*Bt Δ86,87*	This study	Deletion of BT3086 and BT3087
Strain	*Bt VPI-5482*	*Bt Δ86,81*	This study	Deletion of BT3086 and BT4581
Strain	*Bt VPI-5482*	*Bt Δ87,81*	This study	Deletion of BT3087 and BT4581
Strain	*Bt VPI-5482*	*Bt Δ86,87,81*	This study	Deletion of BT3086, BT3087 and BT4581
Strain	*Bt VPI-5482*	*Bt Δ86,87* sfGFP	This study	*Bt Δ86,87* constitutively expressing sfGFP
Strain	*Bt VPI-5482*	*Bt Δ86,81* sfGFP	This study	*Bt Δ86,81* constitutively expressing sfGFP
Strain	*Bt VPI-5482*	*Bt Δ87,81* sfGFP	This study	*Bt Δ87,81* constitutively expressing sfGFP
Strain	*Bt VPI-5482*	*Bt Δ86,87,81* sfGFP	This study	*Bt Δ86,87,81* constitutively expressing sfGFP
Strain	*Bf NCTC-9343*	*Bf mCherry*	Whitaker et al. (40)	*Bf* constitutively expressing mCherry
Plasmid	pNBU2	Λpir pWW3452	Whitaker et al. (40)	Plasmid for insertion of sfGFP into *Bt*
Plasmid	pNBU2	Λpir pWW3515	Whitaker et al. (40)	Plasmid for insertion of mCherry into *Bt*
Plasmid	pLGB13	pLGB13	García-Bayona et al. (41)	Plasmid backbone for *Bt* knockouts
Plasmid	pLGB13	pAB001	This study	Plasmid for the deletion of *BT3086*
Plasmid	pLGB13	pAB002	This study	Plasmid for the deletion of *BT3087*
Plasmid	pLGB13	pAB003	This study	Plasmid for the deletion of *BT3088*
Plasmid	pLGB13	pAB004	This study	Plasmid for the deletion of *BT3089*
Plasmid	pLGB13	pAB005	This study	Plasmid for the deletion of *BT3090*
Plasmid	pLGB13	pAB006	This study	Plasmid for the deletion of *BT3091*
Plasmid	pLGB13	pAB007	This study	Plasmid for the deletion of *BT4581*
Plasmid	pLGB13	pAB008	This study	Plasmid for the deletion of *BT3087* in *ΔBT3086*
Plasmid	pET-29b	pAB010	This study	Plasmid for the recombinant expression of BT3086
Plasmid	pET-29b	pAB011	This study	Plasmid for the recombinant expression of BT3087
Plasmid	pET-29b	pAB012	This study	Plasmid for the recombinant expression of BT4581

Given the functional results of the mutants on growth phenotypes, we wanted to further understand the contributions of each individual GH: BT3086, BT3087, and BT4581 in dextran breakdown. We took an *in vitro* approach to address this question, where we analyzed the degradation products of purified GHs. We first produced the three GHs recombinantly: BT3086 (GH31, α-glucosidase II), BT3087 (GH66, dextranase), and BT4581 (GH97, α-glucosidase), all lacking their signal peptide domains. Individually expressed GH constructs were purified by affinity chromatography and subsequently analyzed by SDS-PAGE (Fig. S1B). We then incubated individual GHs in BMM dextran. We analyzed the resulting digested carbohydrate contents by high-performance anion-exchange chromatography with pulsed amperometric detection (HPAEC-PAD). HPAEC-PAD chromatograms of BMM dextran treated by each of the three enzymes all showed similar patterns of degradations but with quantitative differences (Fig. 1D). BT4581 produced glucose as the sole degradation product, consistent with another previously characterized *Bt* protein that belongs to GH97, SusB of the Sus-like system (42). On the other hand, BT3086 produced glucose and isomaltose as major degradation products, which is again consistent with products formed by exodextranases (GH31) as described previously (33). Finally, BT3087 treatment of dextran produced not only glucose and isomaltose as major degradation products but also other higher MW oligosaccharides, consistent with previous observations of studies involving GH66 endodextranases (33, 35) (Fig. 1D and E). Overall, these data suggest that the enzyme BT3087 is the key GH for the initial debranching of dextran polymers, whereas the enzymes BT3086 and BT4581 play import roles in liberating glucose from dextran—oligosaccharides, which may be subsequently metabolized by *Bt*.

### *Bt* growth dynamics depend on dextran MW

**Fig 2:**
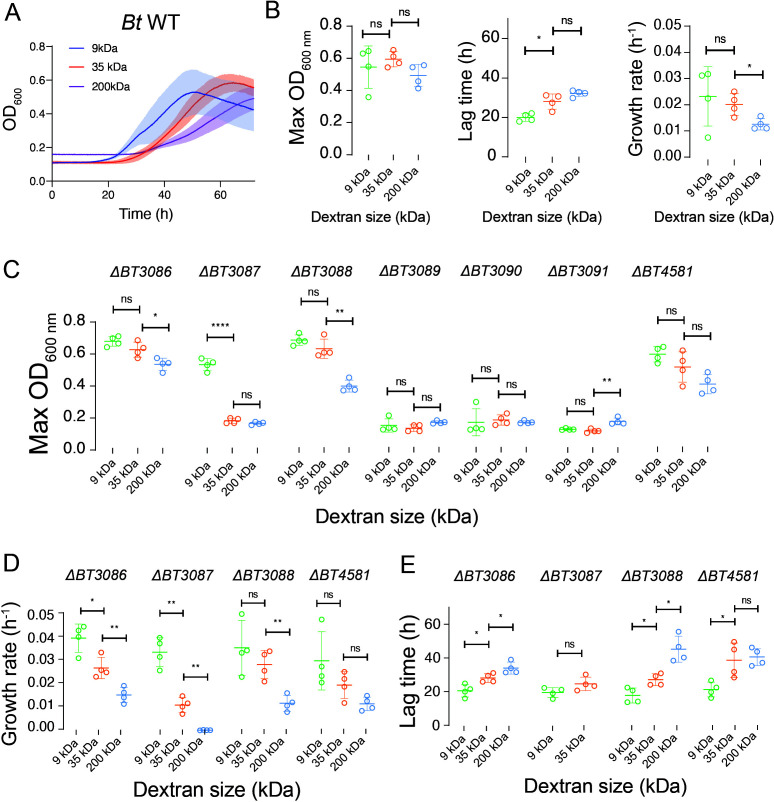
Growth profile quantifications from growth curves of various *Bt* mutants in four different sizes of dextran. (**A**) Growth curves of *Bt* WT in three different sizes of dextran. *N* = 4. (**B**) Extracted parameters from WT *Bt* growth curves in dextran. We quantified the maximum OD, lag phases, and growth rates of *Bt* WT in three different sizes of dextran. We observed a general trend where increasing dextran sizes lead to an increase in lag phase and a decrease in growth rate. (**C**) Maximum OD of each single mutant culture in three different sizes of dextrans. (**D**) Quantified growth rates of individual mutants in three different sizes of dextrans. Mutants that failed to grow were omitted. (**E**) Quantified lag time of individual mutant cultures in three different sizes of dextrans. Mutants that failed to grow were omitted. (**B–E**) All growth experiments: *N* = 4; error bars: standard deviation. Statistical test: *t*-tests were performed between pairs of dextran sizes. Ns (not significant) *P* > 0.05, *P < 0.05, **0.05 < *P* < 0.01, ***0.001 < *P* < .0001, and ****P < 0.0001.

Dextran polysaccharides exist in a wide range of MW and branching that influence its complexity (43, 44). We wondered whether the size of the dextran polymer itself may have an influence on the concerted activity of the Sus-like enzymes and, therefore, on dextran metabolism and subsequent growth dynamics of *Bt*. To explore this possibility, we grew WT *Bt* in dextrans of three different MWs: MW = 9, 35, and 200 kDa (total mass of dextran is equal between conditions). We cultured *Bt* for 3 days while measuring optical density (OD) to eventually generate growth curves (Fig. 2A). We then quantified the maximum OD, lag time, and growth rates of individual cultures (Fig. 2B). We found that the total biomass *Bt* biomass at its maximum value was independent of dextran MW. However, we found important differences in the dynamics of its growth. First, higher dextran MW tends to increase lag time, thereby delaying recovery from the stationary phase. In addition, the higher growth rate of *Bt* is slower in higher MW dextran. Overall, this leads to a delayed growth pattern for *Bt* in higher MW dextran polysaccharide (Fig. 2A).

As we have already cultured all PUL48 mutants supplemented with dextran 35 kDa in test tubes (Fig. 1B) and observed growth defects in mutants lacking BT3087, BT3089, or BT3090, we then wondered whether changes in dextran MW can further impact the growth dynamics of these *Bt* mutants. To explore this question, we measured the growth of PUL48 gene knockouts supplemented with three different MW of dextran polymers with a plate reader (Fig. S2). We first quantified and compared the maximum OD of individual mutant cultures in each of the dextran media (Fig. 2C; Fig. S3A). Mutants lacking the GH BT3087 that could not grow in 35-kDa dextran (Fig. 1B) grew well in 10-kDa dextran. By contrast, we observed that *Bt* mutants lacking BT3089 (SusD), BT3090 (SusC), or BT3091 (SusR) were unable to grow in dextran of any MW. This result suggests that the activity of BT3087 becomes crucial in *Bt* metabolism for growth in high MW dextran. Finally, we observed that the mutant lacking BT3088 (SGBP) also grew in all dextran sizes but with weaker growth at 200 kDa, suggesting that this protein also contributes to dextran utilization at high MW.

We wondered whether these changes in biomass were due to perturbations in growth rates or lag time. We thus measured the impact of dextran MW on the growth rate of the various *Bt* mutants by computing the growth rates of all mutants in different sizes of dextran (Fig. 2D; Fig. S3C). Like the case of *Bt* WT, we observed a trend of decreasing *Bt* growth rates as dextran MW increased. The mutants lacking BT3087 all led to a decrease in growth rate as dextran MW increased, confirming that BT3087 is a crucial GH for supporting *Bt* growth in high MW dextrans (Fig. S3C). The mutant *ΔBT3088* also experienced a sharp decrease in growth rate between growths in dextran 35 and 200 kDa (Fig. 2D), suggesting the importance of this enzyme in the metabolism of high MW dextran polymers by *Bt*. Finally, we quantified the lag phases of all mutants in different MWs of dextran. We observed a general increase in lag time as the size of dextran increases as in WT *Bt*. (Fig. 2E; Fig. S3B). The mutant *ΔBT3088* showed the most drastic increase in lag phase between dextran 35 and 200 kDa.

Overall, our data suggest a dextran size-dependent growth pattern in *Bt*. As dextran MW increases, this leads to a decreased growth rate of *Bt*. We identified three enzymes within PUL48: BT3089 (SusD), BT3090 (SusC), and BT3091 (SusR) that are crucial for *Bt* growth in any dextran size. We also found that BT3087 and BT3088 (SGBP) promote *Bt* dextran metabolism at high MW. Interestingly, our data also suggest that large dextran oligosaccharides of 9 kDa can be metabolized by *Bt* without the aid of BT3087.

### Tracking dextran binding using fluorescent polymers

**Fig 3:**
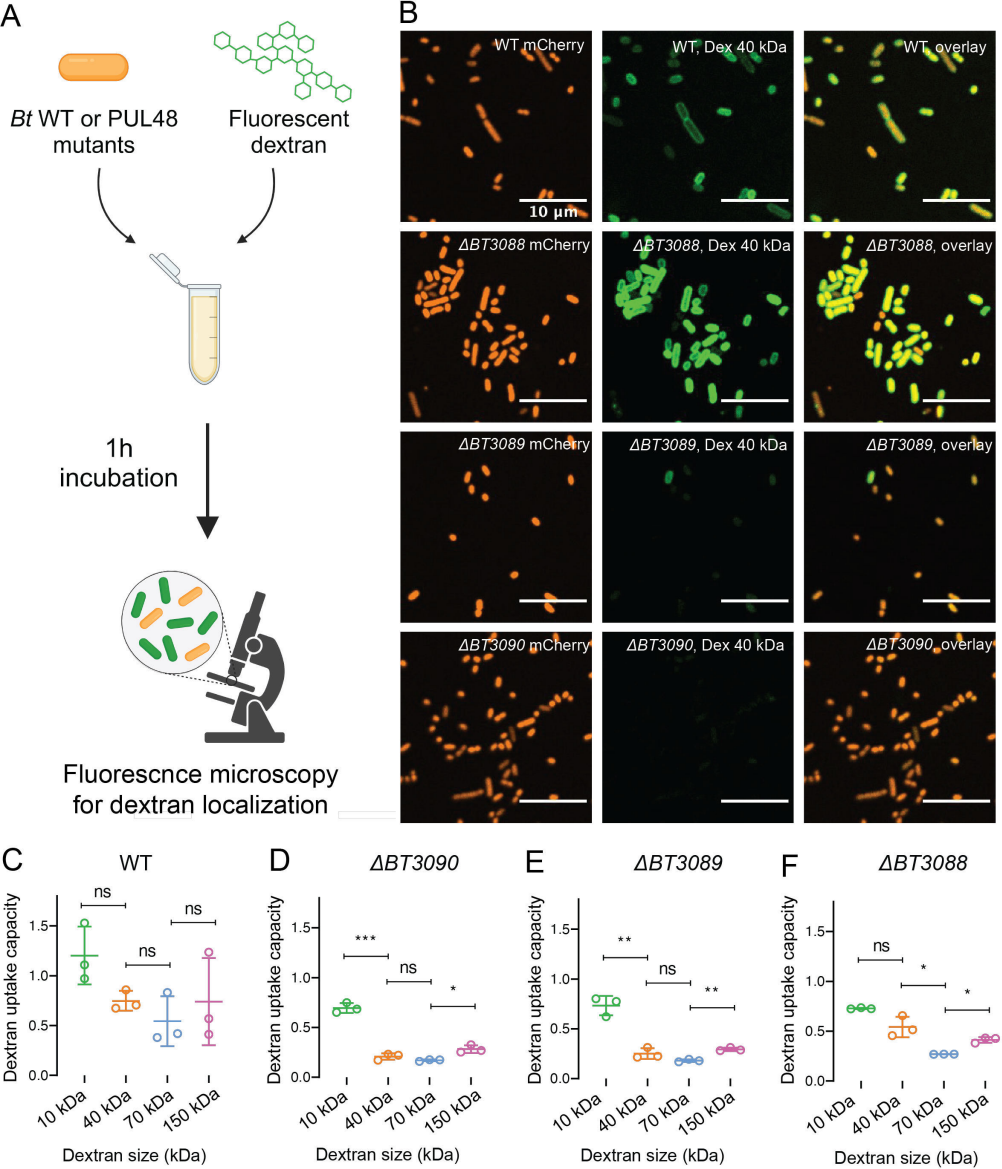
Fluorescent dextran localization to *Bt* cells. (**A**) Schematic of experimental set-up. *Bt* cells constitutively expressing mCherry are incubated with fluorescein dextran of various MWs for 1 hour, washed, and imaged by fluorescence microscopy. (**B**) Fluorescence microscopy images of *Bt* WT along with various mutants constitutively expressing mChery incubated in dextran 40 kDa conjugated to fluorescein. Red channel corresponds to *Bt* cells, green channel corresponds to dextran, and the overlay corresponds to localization of dextran to *Bt* cells. (**C**) The binding capacity of various *Bt* mutants to varying sizes of dextran molecules. The binding capacity is defined as the ratio between green and red fluorescence signals per individual cells. The ratio for individual cells within a field of view were then averaged and plotted. For all fluorescein dextran-binding experiments, *N = 3*. Statistical test: *t*-tests were performed between pairs of dextran sizes. *P < 0.05, **0.05 < *P* < 0.01, ***0.001 < *P* < 0.0001, and ****P < 0.0001.

To further investigate the mechanisms of binding and import of polysaccharides of various sizes, we designed a fluorescence microscopy-based assay for dextran localization at the surface of *Bt* cells (Fig. 3A). We cultured *Bt* cells all expressing the fluorescent protein mCherry constitutively in the following backgrounds: WT, *ΔBT3088* (SGBP*-*), *ΔBT3089* (*susD-*), and *ΔBT3090* (*susC-*). We grew these strains in BMM containing equal amounts of dextran and glucose. We then diluted the cells in phosphate buffered saline (PBS) supplemented with fluorescein-conjugated dextran of various sizes. The solution was then incubated aerobically for 1 hour, washed, and imaged by high-resolution fluorescence microscopy. All samples were imaged in both the red and green fluorescence channels, which correspond to *Bt* cells and fluorescent dextrans respectively (Fig. 3B; Fig. S4). Microscopy images of *Bt* incubated with 40-kDa dextran fluorescein showed a strong localization of green fluorescence signal to WT and *ΔBT3088* cells but not *ΔBT3089* and *ΔBT3090* cells. These results suggest that mutants lacking SusD or SusC lose the ability to bind 40-kDa dextran on the cell surface, potentially explaining their inability to grow in dextran (Fig. 2C).

We then measured the dextran-binding capacity of each *Bt* strain by quantifying dextran fluorescence per cell normalized to the mCherry fluorescence of the same cell. We then average this for all cells. In the case of *Bt* WT, we could not distinguish differences in the dextran-binding capacity across the four different sizes of dextran polymers tested (Fig. 3C). We then performed the same analysis on PUL48 mutants (Fig. 3D to F). First, we observed that the deletion of BT3089 (SusD) or BT3090 (SusC) led to a significant decrease in green fluorescence upon treatment with dextran polymers of MWs higher than 10 kDa (Fig. 3D and E), suggesting that these two enzymes are important for the specific localization of dextran molecules to the cell membrane of *Bt*. Interestingly, the deletion of BT3088 (SGBP) led to a decrease in green fluorescence localization upon treatment with dextran polymers greater than 40 kDa in MW (Fig. 3F), suggesting that this protein is important in *Bt* dextran binding in a size-dependent manner. Finally, we observed that all three deletions led to no significant changes in green fluorescence upon treatment with 10-kDa dextran compared to WT (Fig. S5), suggesting that this dextran binds to *Bt*’s cell membrane without the aid of the PUL48 machinery. Together, these data suggest that the PUL48 enzymes BT3088, BT3089, and BT3090 play a crucial role in the dextran binding by *Bt* and that the MW of dextrans itself does not have an impact on the binding capability by WT *Bt* cells.

### PUL48 and dextran size define the extent of nutrient sharing by *Bt*

**Fig 4:**
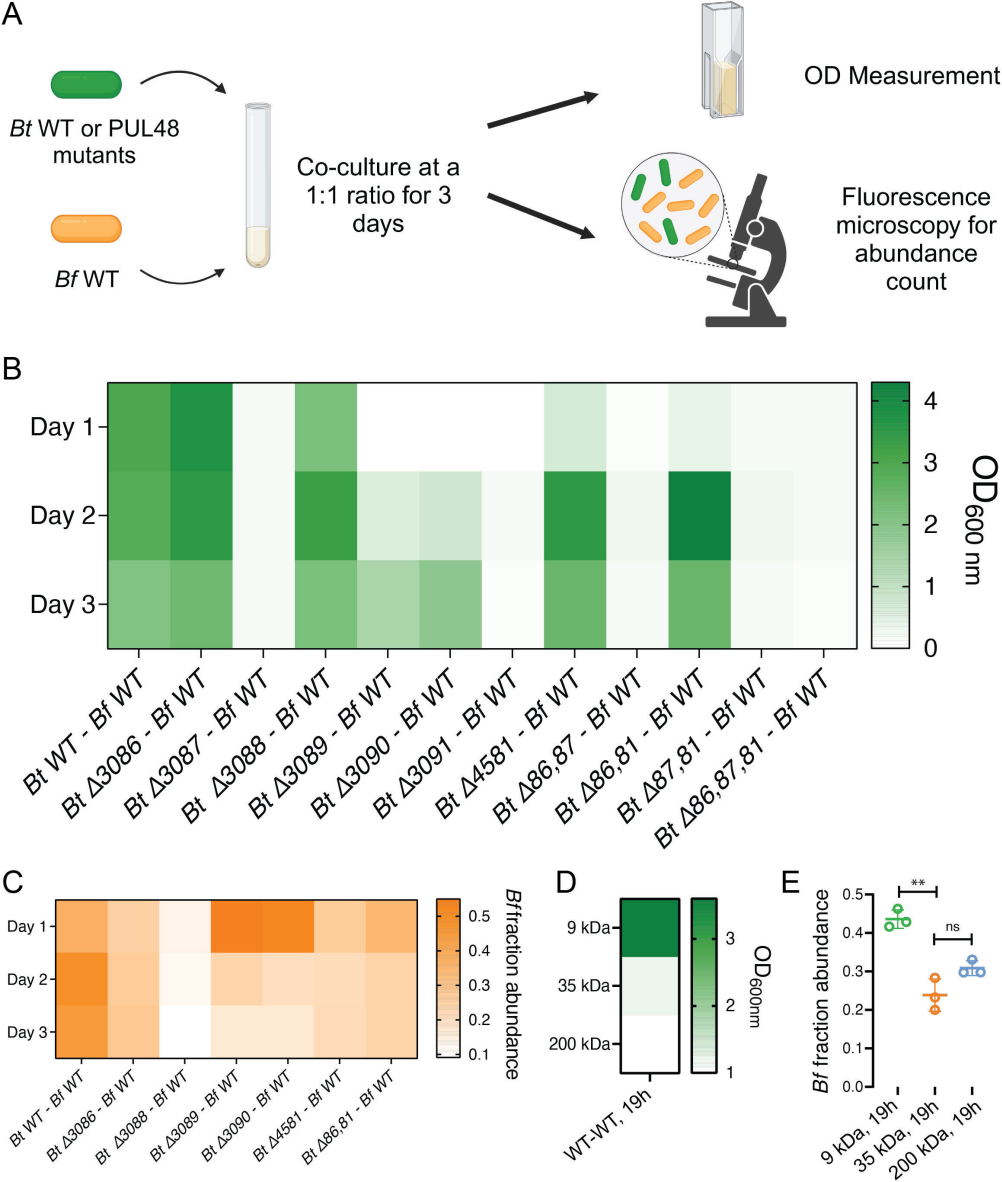
PUL48 proteins contribute to the extent of nutrient sharing from *Bt* to *Bf* in dextran. (**A**) Schematic of experimental set-up. *Bt* WT or mutants expressing sfGFP are mixed with *Bf* WT at a 1:1 ratio. OD measurements and fluorescence microscopy were then performed daily to study coculture growth and *Bf* abundance. (**B**) Co-culture growths of *Bt* WT or PUL48 mutants with *Bf* in dextran. We monitored the OD daily over the course of 3 days. (**C**) *Bf* fraction abundance from competition experiments. *Bt* strains that failed to grow as monocultures have been omitted from co-culture analysis. Our results indicate that the loss of any one enzyme associated with PUL48 led to a decrease in nutrient sharing to *Bf* in comparison to WT levels. (**D**) Co-culture growths of *Bt* WT with *Bf* in three different sizes of dextran polymers. We monitored the OD of individual cultures upon 19 hours of growth. (**E**) *Bf* fraction abundance from *Bt* WT to *Bf* WT co-culture experiments in three different sizes of dextran upon 19 hours of growth.

We previously demonstrated that *Bt* can share by-products of dextran metabolism with *Bacteroides fragilis* (*Bf*), ultimately guiding community composition and biofilm spatial organization (30). We thus wondered whether individual PUL48 proteins may influence the nature of nutrient sharing by *Bt* to other members of a community, including in a MW-dependent manner. To answer this question, we grew *Bt-Bf* each constitutively expressing distinct fluorescent proteins in competition and monitored the relative fraction of each species in the community using fluorescence microscopy (Fig. 4A). We first quantified the overall biomass (via OD) of the co-cultures after 3 days (Fig. 4B). The growth patterns in co-cultures were identical to the *Bt* monocultures in dextran from Fig. 1C: co-cultures containing mutants that lack the GH BT3087 or the regulator BT3091 failed to grow. We then measured the abundance of *Bf* in each co-culture by sampling each co-culture upon 1, 2, and 3 days of growth under anaerobic conditions. When computing the *Bf* fraction abundance by computing the ratio between mCherry- and sfGFP-positive cells (Fig. 4C), we found that with when co-cultured with WT *Bt*, the fraction abundance of *Bf* overtime is maintained at 50% (30). This ratio, however, decreases in all *Bt* PUL48 mutants that have the ability to grow in dextran: *ΔBT3086*, *ΔBT3088*, *ΔBT3089*, *ΔBT3090*, *ΔBT4581*, and *Δ86,81*. These results suggest that the deletion of any one of these proteins potentially leads to a reduction in the availability of metabolic by-products like glucose, which ultimately leads to a reduction of nutrient availability for glycolysis in *Bt*.

Finally, based on different metabolic rates of *Bt*, we hypothesized that the size of polysaccharide polymers modulates nutrient sharing to impact community composition. To address this question, we competed *Bt* WT with *Bf* WT at a 1:1 ratio in three different sizes of dextran. We sampled these co-cultures after overnight growth (Fig. 4D) and measured OD along with the fraction abundance of *Bf* by fluorescence microscopy (Fig. 4E). First, we observed that the final OD of co-cultures decreases as the size of dextran polymers increased, consistent with our *Bt* monocultures (Fig. 2A). We found that co-cultures grown in higher MW dextran (35 and 200 kDa) have a decrease in *Bf* relative abundance (Fig. 4E). These results suggest that the size of polysaccharides impacts *Bt*’s growth rate, where reduced *Bt* growth impacts the amount of glucose metabolites generated at a given time. Increasing dextran MW reduced glucose production rate and secretion that may decrease *Bf* growth. Altogether, these results show that polysaccharide size can impact nutrient sharing in microbial communities, leading to changes in community compositions.

## DISCUSSION

During cycles of dietary shifts, changes in glycan composition greatly contribute to defining microbiota composition (5). PUL functions are quite specific in processing glycans. However, within each polysaccharide family, there exist a variety of molecular structures that introduce another layer of diversity, but how this impacts the functions of PULs has been largely overlooked. To comprehensively understand how diet modulates commensal species metabolism that ultimately shapes our gut microbiota, it is crucial to understand how this physical complexity impacts their metabolic pathways.

We identified a general trend of decreasing *Bt* growth rates as dextran MW increased. We attribute this decrease to the increased structural complexity of dextran polymers through branching, which reduces the processivity of PUL48 (45). As structural complexity increases, the liberation of free oligosaccharides and glucose from dextran may become increasingly difficult for *Bt*. This leads to a reduced pool of oligosaccharides and glucose, leading to an overall decrease in growth rate in dextran polymers of higher MW. At the molecular level, we identified two enzymes whose contribution becomes crucial at high MW: the dextranase BT3087 and the SGBP protein BT3088. We found a cut-off at 9 kDa for BT3087. This differs from the SusC-SusD complexes of the Sus system and levan utilisome of *Bt*, which selectively imports only short maltoolisaccharides and fructooligosaccharides with degrees of polymerization of 5–16 (46) and 5–15 (47), respectively, which are much smaller in comparison to the dextran of 9 kDa in size. This may be attributed to the overall structure of this particular dextran polymer as it is known that dextran 2–10 kDa exhibits properties of an expandable coil, with minimal branching (44), which potentially allow native dextran to pass through the importer BT3090 (SusC) without the aid of BT3087. Another protein that we found to contribute to dextran utilization in a size-dependent manner is BT3088 (SGBP). The exact roles of SGBP proteins in polysaccharide utilization have remained unclear (46). However, we observed that the deletion of the SGBP BT3088 led to a size-dependent growth defect in *Bt* when cultured in dextran polymers greater than 35 kDa in size. Our results suggest that BT3088 likely plays an important role in *Bt’s* binding to complex dextran polymers or oligosaccharides generated by complex dextran metabolism, which subsequently facilitates the import of extra oligosaccharides into the cell.

Leveraging cryogenic electron microscopy (cryo-EM) to analyze PULs, a previous work has shown that in fact, outer membrane-associated PUL proteins form a stable utilisome complex and act as one unit (34). Our results also show that dextran surface binding is a key function in its metabolism and depends on surface-exposed GHs. SusC-like and SusD-like proteins play important roles in binding and uptake. Cryo-EM data also suggested the role of SGBPs in the binding of free glycans and their potential interactions with surface-associated GHs, potentially increasing glycan concentration local to the active site of the utilisome unit (34). Our fluorescein dextran-binding data for *Δ*BT3088 further confirm that the SGBP BT3088 plays an important role in *Bt* dextran binding. In particular, SGBPs become increasingly important for *Bt* binding of dextran greater than 70 kDa in size. These results may be potentially explained by the increasing importance of SGBP activity for the binding and guiding of complex, high MW dextran to the active site of the dextran utilisome complex. Our observations are reminiscent of adhesion of *Ruminococcus bromii*, a common member of the gut microbiota that also utilize starch, which expresses surface enzymes that facilitate adhesion to starch molecules (48).

We then sort to understand the contribution of PUL enzymes and polysaccharide MW on species interactions and abundance at the community level. It is known that in *B. ovatus (Bo)*, the extent of nutrient sharing of metabolite by-products through xylan metabolism to *Bifidobacterium adolescentis* is dependent on the structural complexity of the polysaccharide. In particular, the differing numbers and types of sugar side chains attached to the different classes of xylan polymers impact the complexity of resulting metabolic by-products released by *Bo*, therefore limiting nutrient sharing to neighboring strains based on the availability of enzymes required for the degradation and utilization of the released metabolic by-products of increased structural complexity (49). Here, we observed that the deletion of any one protein within PUL48 led to a decrease in *Bf* abundance in a two-species community. Such decrease in *Bf* abundance is likely a result of the reduced availability of the public good glucose. The deletion of BT3086 or BT4581, two α-glucosidases that are localized in the periplasm, reduces the formation of glucose from oligosaccharides. As BT3087 also generates glucose as a product, extracellular glucose may be preferentially imported by *Bt*, reducing the overall pool of public goods available for *Bf* to exploit. As the deletion of BT3088 also led to a significant decrease in *Bf* population, BT3088 also likely contributes to the uptake of oligosaccharides in *Bt*, hence also leading to the import of extracellular glucose and reducing nutrient availability to *Bf*.

Previous studies have demonstrated that galactans of differing structural complexities have an impact on the species-specific fermentation by gut microbes, which leads to changes in overall microbiota composition (50). More precisely, it had been previously demonstrated with blackberry polysaccharides that the MW of the polysaccharides impacts the rate of fermentation and ultimately the composition of the overall microbial community (51). Our data also suggest a potential mechanism where polysaccharide size impacts nutrient sharing in microbial communities through defining the growth rate of the utilizer and restricting the amount of public goods generated and shared to the non-utilizer, which ultimately lead to an increasing delay in growth and colonization by the non-utilizer.

Overall, our work provides a novel mechanism of commensal selection based on the complexity of dietary polysaccharides. Our observations imply that at equal molecular composition and concentration, a seemingly identical dietary composition can lead to distinct community composition depending on the structure of polysaccharide molecules it contains. As many other dietary fibers also likely exist in large ranges of MW, understanding how MW impacts utilization by gut microbe may lead to the potential design of next-generation precision prebiotics.

## MATERIALS AND METHODS

### Strains and culture media

*Bt* VPI-5482 WT, *Bt* VPI-5482 sfGFP, and *Bf* NCTC-9343 mCherry, along with all *Bt* VPI-5482 PUL48 knockout mutants (Table 1), were used for experiments in this work. TYG medium and BMM supplemented with the indicated carbon source were used in all experiments.

For TYG medium, 10-g tryptone, 5-g yeast extract, 2.5-g d-glucose, and 0.5-g l-cysteine were dissolved in 465 mL of ddH_2_O. The resulting solution was then immediately autoclaved and allowed to cool down to room temperature. In the meantime, salt solution A was prepared by mixing 0.26 g of CaCl_2_ and 0.48 g of MgSO_4_ in 300 mL of ddH_2_O until fully dissolved where 500 mL of ddH_2_O was then added. One gram of KH_2_PO, 1 g of K_2_HPO_4_, and 2 g of NaCl were then added to the solution and stirred at room temperature until all salts were fully dissolved followed by the addition of 200 mL of ddH_2_O to top up the volume. The salt solution was then stored at 4°C for future use. A 10% (wt/vol) NaHCO_3_ solution was prepared by dissolving 50 g of NaHCO_3_ into 500 mL of ddH_2_O followed by filter sterilization with a 0.22-µm filter. A 1.9-mM hematin–0.2-M histidine solution was prepared by dissolving 60.18 mg of hematin in 1 mL of 1-M NaOH until fully solubilized and then neutralized with 1 mL of 1-M HCl. In a separate beaker, 1.55 g of l-histidine was dissolved in 48 mL of ddH_2_O. The histidine solution was then combined with the hematin solution, filter-sterilized with a 0.22-µm filter, and stored at 4°C for future usage. Five milliliters of hematin-histidine solution and 10 mL of 10% NaHCO_3_ solution were added to the TYG base medium with a 0.22-µm filter. With a separate 0.22-µm filter, 20 mL of salt solution A was then added. The resulting complete TYG medium was then stored at 4°C for future usage.

The BMM medium was prepared as previously described (52, 53). Briefly, a 10× medium stock containing 1-M KH_2_PO_4_, 150-mM NaCl, and 85-mM (NH_4_)_2_SO_4_ was first prepared and adjusted to a pH of 7.2. Solutions of 1-mg/mL vitamin K_3_, 0.4-mg/mL FeSO_4_, 0.1-M MgCl_2_, 0.8% (wt/vol) CaCl_2_, 0.01-mg/mL vitamin B_12_, and 1.9-mM hematin–0.2-M histidine were prepared separately. To prepare 100 mL of BMM, 10 mL of the 10× salts were mixed with 0.1 g of l-cysteine, 50 µL of vitamin B_12_ solution, and 100 µL of each vitamin K_3_, FeSO_4_, MgCl_2_, CaCl_2_, and hematin-histidine solution. All carbon sources were added to a final concentration of 5 mg/mL. The media were filter-sterilized using a 0.22-µm filter unit and degassed in the anaerobic chamber overnight.

All bacteria were cultured at 37°C under anaerobic conditions in a vinyl anaerobic chamber (COY) inflated with a gas mix of approximately 5% carbon dioxide, 90% nitrogen, and 5% hydrogen (CarbaGas). Bacteria were first streaked on brain heart infusion plates (Sigma-Aldrich) containing 10% sheep blood (TCS BioSciences). Single colonies were then inoculated in BMM and grown overnight.

### Recombinant expression and purification of *Bt* GHs

The amino acid sequences of the GHs BT3086, BT3087, and BT4581 were collected from the *Bt* VPI-5482 genome. The amino acid sequences of each GH were first analyzed by SignalP (54) in order to identify signal peptide and potential transmembrane domains of the GHs. Each of these genes was then amplified by PCR without the corresponding signal peptide regions and cloned into the expression vector pET29b containing an N-terminal 10× His-tag. The vector was then transformed into *Escherichia coli* XL10 gold for plasmid production and storage. The resulting plasmid was then transformed into *E. coli* BL-21, where then a 50-mL overnight culture is grown in Lysogeny Broth (LB) kanamycin (Kan). The overnight culture was then transferred into 2 L of LB Kan and grown until the OD of 0.6 at 37°C. The cells were then induced with 1-M IPTG and cultured overnight at 19°C. The culture was then pelleted, washed, lysed, and centrifuged at 48,254.4 rcf to remove membrane material. The resulting supernatant was then purified using an affinity Ni-NTA column, eluted in 1× PBS, and stored at −80°C until usage. The resulting purified proteins were then analyzed by SDS-PAGE to confirm expression and identity.

### GHs activity analysis by HPAEC-PAD

Twenty microliters of 1-mg/mL purified GHs: BT3086, BT3087, or BT4581 (stocks equivalent to 10.3-µM BT3086, 14.6-µM BT3087, or 12.1-µM BT4581) were added to 1 mL of BMM dextran MW 9,000–11,000. The mixture was then incubated overnight anaerobically at 37°C. Cation and anion exchange chromatography with AmberChrom resins (Sigma-Aldrich) was then used to remove protein and ions from the medium, followed by filtering through a 0.22-µm sterilization filter prior to analysis. The resulting flow throughs were then analyzed by HPAEC-PAD. The sugars were separated on a Dionex CarboPac PA-20 column from Thermo Fisher Scientific with the following eluent: (i) 100-mM NaOH and (ii) 150-mM NaOH and 500-mM sodium acetate at a flow rate of 0.4 mL/minute. The gradient was 0–15 minute, 100% A (monosaccharide elution); 15–26 minute, gradient to 10% A and 90% B (maltooligosaccharide elution); 26–36 minute, 10% A and 90% B (column wash step); and 36–46 minute, step to 100% A (column re-equilibration). Peaks were identified by co-elution with known glucose, isomaltose standards using the Chromeleon software. The HPAEC-PAD data were then exported and replotted with the graphing software GraphPad Prism 8, where the area under the curve of peaks of corresponding samples was calculated.

### In-frame deletion knockouts in *Bt*

*Bt* knockouts were generated as previously described (41). Briefly, 700-bp upstream and downstream of each gene of interest were amplified from *Bt* WT by PCR (with primers desribed in Table 2). The resulting PCR products were then analyzed on a 1% agarose gel and subsequently gel-extracted. The knockout plasmid pLGB13 was then restriction-digested with PstI and BamHI, where the upstream fraction, downstream fraction, and digested pLGB13 were assembled via Gibson Assembly. The assembled plasmid was then transformed into the donor strain *E. coli S17*. The donor strains carrying the knockout plasmids were then grown to an OD of 1–1.2 in LB-Amp 100. At the same time, *Bt* strains were grown anaerobically to an OD of roughly 0.1. The donor culture was then washed with PBS, pelleted, and mixed with the *Bt* culture. The culture is then puddled onto BHI plates supplemented with 10% sheep blood and dried for conjugation aerobically overnight at 37°C. The resulting carpet is then resuspended in TYG medium and restreaked onto BHI sheep blood plates with gentamycin (Gm) 200 µg/mL and erythromycin (Er) 20 µg/µL and grown anaerobically for 30 hours. The resulting colonies were picked and restreaked directly onto another BHI-sheep blood plate containing Gm/Er for another 30 hours anaerobically. Resulting single colonies were then picked and restreaked onto BHI-sheep blood plate containing 0.1 µg/mL of anhydrotetracycline for counter-selection and grown anaerobically for 30 hours. Resulting single colonies were again picked, where colony PCR was then performed, and the resulting PCR product was sequenced to confirm whether the knockout was successful.

**TABLE 2:** List of primers used for this study

Primer number	Primer name	Description	Primer sequence (5′ to 3′)
AB001	3086 KO up FOR	Knockout BT3086 in WT	ATTAGCATTATGAGTCCCGAAGGAACATGGTAC
AB002	3086 KO up REV	Knockout BT3086 in WT	CTTGAACTCAACGCCTACA
AB003	3086 KO down FOR	Knockout BT3086 in WT	GGCGTTGAGTTCAAGACCTATCGTTTTTAGTTATTCCG
AB004	3086 KO down REV	Knockout BT3086 in WT	CTTGATATCGAATTCAAAGCCGTCCTGTATGAAAA
AB005	3087KO up FOR	Knockout BT3087 in WT	ATTAGCATTATGAGTATAATCACGGCTTTCCGTT
AB006	3087 KO up REV	Knockout BT3087 in WT	GGCAGTTGCAACGGAATA
AB007	3087 KO down FOR	Knockout BT3087 in WT	TCCGTTGCAACTGCCCTTGAATACTTGTTTATTGTTTTACAATTG
AB008	3087 KO down REV	Knockout BT3087 in WT	CTTGATATCGAATTCGGCGATAATGGCTGGGG
AB009	3088 KO up FOR	Knockout BT3088 in WT	ATTAGCATTATGAGTCTTCAATGCCCCGAAACA
AB010	3088 KO up REV	Knockout BT3088 in WT	ACAAGTATTCAAGATGAAGAAGATAATATA
AB011	3088 KO down FOR	Knockout BT3088 in WT	ATCTTGAATACTTGTCATATGATCTGTCTAAATGAATGATTG
AB012	3088 KO down REV	Knockout BT3088 in WT	CTTGATATCGAATTCCGGCAGTACGGAATACGA
AB013	3089 KO up FOR	Knockout BT3089 in WT	ATTAGCATTATGAGTGCCGTAGTTCTTCTCCG
AB014	3089 KO up REV	Knockout BT3089 in WT	TTGCTTTTACAATCATTCATTTAGAC
AB015	3089 KO down FOR	Knockout BT3089 in WT	TGATTGTAAAAGCAATTTCTTCCTCCTGATTTAAAAGTT
AB016	3089 KO down REV	Knockout BT3089 in WT	CTTGATATCGAATTCCAATTCATACGTGACCATGC
AB017	3090 KO up FOR	Knockout BT3090 in WT	ATTAGCATTATGAGTGATTGGGCGCCAGCG
AB018	3090 KO up REV	Knockout BT3090 in WT	ATCAGGAGGAAGAAAATGAAAAAGAAAC
AB019	3090 KO down FOR	Knockout BT3090 in WT	TTTCTTCCTCCTGATGTACATCAATTTAAAGTTAATATTAGGATTACT
AB020	3090 KO down REV	Knockout BT3090 in WT	CTTGATATCGAATTCGACTCCAATTCGCAACTGAA
AB021	3091 KO up FOR	Knockout BT3091 in WT	ATTAGCATTATGAGTTTTAATGGTTACATTGTCCCC
AB022	3091 KO up REV	Knockout BT3091 in WT	GAAATCCACTACTTTTTTAGCAC
AB023	3091 KO down FOR	Knockout BT3091 in WT	AAAGTAGTGGATTTCAGCGCCATATGTATTATATCTGC
AB024	3091 KO down REV	Knockout BT3091 in WT	CTTGATATCGAATTCTATAAAAATATTGATGGAGCAATGGC
AB025	4581 KO up FOR	Knockout BT4581 in WT	ATTAGCATTATGAGTAGAAATATATCAGCATTAAACTTCTCC
AB026	4581 KO up REV	Knockout BT4581 in WT	GATATAAATGAATTAGTTAATAATCATATGGC
AB027	4581 KO down FOR	Knockout BT4581 in WT	TAATTCATTTATATCGACGCGACTAAAACGATTGTTC
AB028	4581 KO down REV	Knockout BT4581 in WT	CTTGATATCGAATTCACCGGAGTACGTCCCCAT
AB046	pET29_BT3086 FOR	Expression of BT3086	ATCGAATTCGGGATCCGAAAACGCAAAAAGTATATGTACC
AB047	pET29_BT3086 REV	Expression of BT3086	GTGGTGGTGGTGCTCGAGTCATAAACGCAGGGAAATCC
AB050	pET29_BT3087 FOR	Expression of BT3087	GATCGAATTCGGGATCCTGCAGCGACGATCATGA
AB051	pET29_BT3087 REV	Expression of BT3087	GTGGTGGTGGTGCTCGAGTTATTCAGCTACAATCATTGTCCA
AB052	pET29_BT4581 FOR	Expression of BT4581	GGATCGAATTCGGGATCCGAAAGTATCACTTCTCCTGACG
AB053	pET29_BT4581 REV	Expression of BT4581	TGGTGGTGGTGGTGCTCGAGTCATTTCCATTTCTTAATTGATTTCC
AB095	3086 3087 KO up FOR	Knockout BT3087 in BT3086 background	GATTAGCATTATGAGTAGAAATATGAAGGCAACCGT
AB096	3086 3087 KO up REV	Knockout BT3087 in BT3086 background	AAACAAGTATTCAAGGGCAGTTGCAACGGAAT
AB097	3086 3087 KO down FOR	Knockout BT3087 in BT3086 background	CTTGAATACTTGTTTATTGTTTTACAATTG
AB098	3086 3087 KO down REV	Knockout BT3087 in BT3086 background	CTTGATATCGAATTCGGCGATAATGGCTGGG

### Insertion of sfGFP and mCherry into *Bt* and *Bf*

Fluorescent strains of *Bt* and *Bf* were generated as described previously (40). Briefly, the plasmids pWW3452 (GFP) and pWW3515 (mCherry) were transformed into the donor strain *E. coli S17*. The donor was then grown to an OD of 1–1.2 in LB-Amp 100. At the same time, *Bt* or *Bf* strains were grown anaerobically to an OD of roughly 0.1 (for *Bf*, OD 0.01). The donor culture was then washed with PBS, pelleted, and mixed with either the *Bt* or *Bf* culture. The culture is then puddled onto BHI plates supplemented with 10% sheep blood and dried for conjugation aerobically overnight at 37°C. The resulting carpet is then resuspended in TYG medium and restreaked onto BHI sheep blood plates with Gm 200 µg/mL and Er 20 µg/µL and grown anaerobically for 30 hours. The resulting colonies were picked and restreaked directly onto another BHI-sheep blood plate containing Gm/Er for another 30 hours anaerobically. The resulting colonies were then resuspended and screened for fluorescent properties on the confocal fluorescence microscope.

### Test tube mutant growths and competitions

For initial growth experiments, *Bt* and various mutants were cultured in BMM supplied with 5 mg/mL of D(+)-glucose (Carl Roth) overnight anaerobically. The resulting cultures were then pelleted by centrifugation and washed twice with PBS. The pellets were then resuspended in PBS, where the OD was measured. The various *Bt* strains were then diluted to a final OD of 0.1 or 0.01 in BMM dextran (MW = 35 kDa). The cultures were then grown anaerobically for 3 days, where the OD of individual mutants was measured after Days 1, 2, and 3. For *Bt* mutant, competition experiments with *Bf*, *Bt*, and *Bf* were cultured separately in 1 mL of BMM glucose overnight. The resulting cultures were then pelleted by centrifugation and washed twice with PBS. The pellets were then resuspended in PBS, where the OD was measured. *Bt* and *Bf* were then mixed at a 1:1 ratio to an initial OD of 0.1 in each of the carbon sources tested. The cultures were then grown anaerobically for 3 days. A microscopy approach was then used to quantify the relative abundances of *Bt* and *Bf* in these cultures. To achieve this, the cultures were mixed well, and 10 µL of each cell culture was sampled on Days 1, 2, and 3. The cultures were then diluted in PBS and incubated at aerobic conditions. The samples were then spotted onto a glass coverslip and covered by a thin agarose pad prior to fluorescence imaging by fluorescence microscopy. For each sample and replicate, three image frames across the agar pad were recorded. The relative abundance of sfGFP- and mCherry-expressing cells in each sample was then quantified using the “analyze particle” function in Fiji and averaged across the three frames.

### Growth curves analysis of *Bt*

*Bt* and various mutants were cultured in BMM supplied with 5 mg/mL of D(+)-glucose overnight anaerobically. The resulting cultures were then pelleted by centrifugation and washed twice with PBS. The pellets were then resuspended in PBS, where the OD was measured. The various *Bt* strains were then diluted to a final OD of 0.1 in BMM dextran of the corresponding MWs (Sigma-Aldrich) in a clear COSTAR 96-well plate with a final volume of medium at 200 µL. The plate is then completely sealed with parafilm, moved into the Tecan Safire 2 plate reader, and grown anaerobically at 37°C: settings: kinetic, 5-minute cycles, 875 cycles; shake 10 seconds prior to each analysis cycle. The resulting data were then rearranged by a house-built Python script, where all growth curves were then plotted by the graphing software GraphPad Prism 8. For further analysis for the lag time, maximum OD, and maximum growth rate of each condition, the file of rearranged data was analyzed using Pyphe growth curves (55).

### *Bt* binding to fluorescently labeled dextran

*Bt* WT and various mutants containing mCherry were cultured in BMM supplied with 2.5 mg/mL of D(+)-glucose and 2.5 mg/mL of dextran 35 kDa overnight anaerobically. The resulting cultures were then pelleted by centrifugation and washed twice with PBS. The pellets were then resuspended in 1× PBS, where the OD was measured. We then diluted the cells to a final OD of 0.1 in PBS and added fluorescein-conjugated dextran of various sizes (Sigma-Aldrich) to the solution at a final concentration of 1 mg/mL. We then incubated the solution aerobically for 1 hour, and the cells were then washed 3× in 1× PBS. Resulting cells were then resuspended in 1× PBS and imaged by fluorescence confocal microscopy. For each sample and replicate, five image frames across the agar pad were recorded. The relative abundance of mCherry- and sfGFP-expressing cells of signal corresponding to dextran binding and import was recorded for each single cell within each sample using the “analyze particle” function in Fiji. The ratio of sfGFP:mCherry was then quantified for every individual cell within an experiment and averaged per experiment.

### Microscopy

Imaging was performed using a Nikon Eclipse Ti2-E inverted microscope coupled with a Yokogawa CSU W2 confocal spinning disk unit and equipped with a Prime 95B sCMOS camera (Photometrics). The 40× Plan APO objective with a numeric aperture of 0.9 was used for all imaging. Fiji was then used to display the images.

## SUPPLEMENTAL MATERIAL

Supplemental figures mbio.02599-23-s0001.docx Fig. S1 to S5.

ASM does not own the copyrights to Supplemental Material that may be linked to, or accessed through, an article. The authors have granted ASM a non-exclusive, world-wide license to publish the Supplemental Material files. Please contact the corresponding author directly for reuse.
