# Supplementary material for: Bacteroides thetaiotaomicron metabolic activity decreases with polysaccharide molecular weight
Source: mBio. 2024 Feb 20;15(3):e02599-23. doi: 10.1128/mbio.02599-23 (PMC10936149; doi:10.1128/mbio.02599-23)
Supplement: Supplemental figures — Fig. S1 to S5. [file mbio.02599-23-s0001.docx]

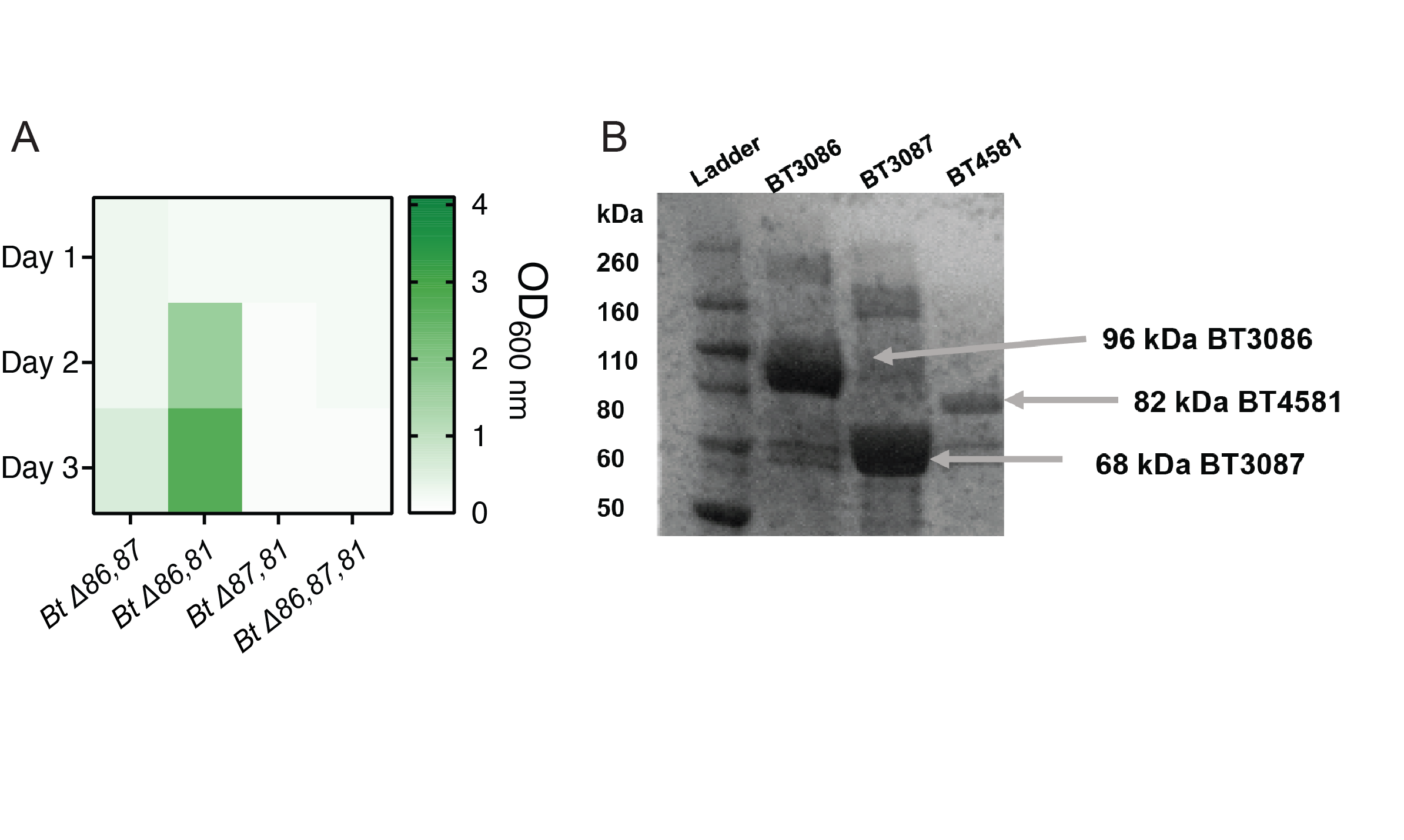


**Figure S1: Involvement of GHs BT3086, BT3087 and BT4581 in *Bt* dextran metabolism.** (A) Monoculture growths of *Bt* double and triple GH knockout mutants. We measured the optical density (OD) of individual cultures everyday over the course of 3 days (B) SDS-PAGE analysis of recombinantly expressed BT3086. BT3087 and BT4581 upon purification by Ni-NTA affinity chromatography.


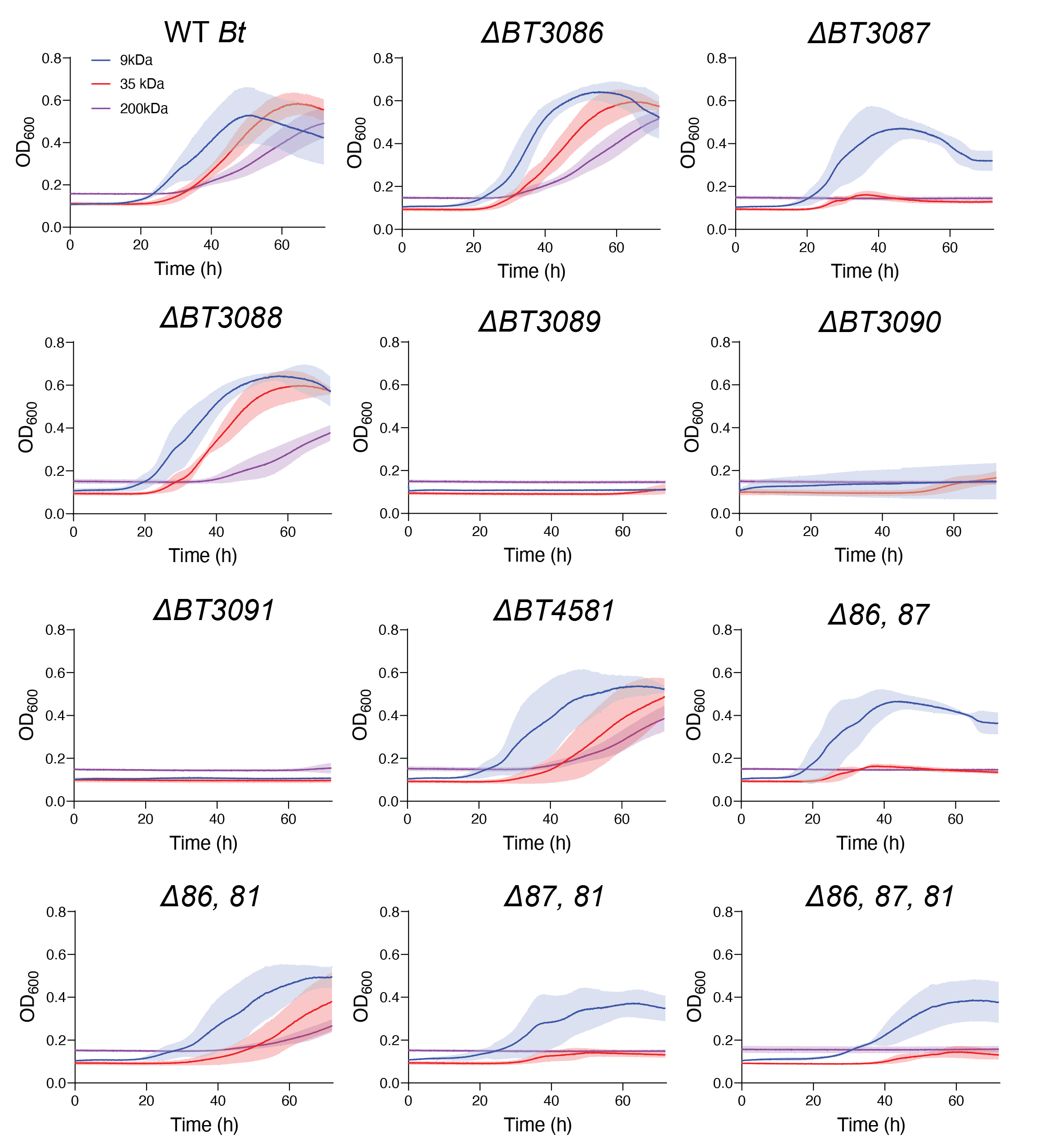


**Figure S2: Growth curves of *Bt* WT and various PUL48 mutants in 3 different sizes of dextran over the course of 3 days.** *N = 4,* shaded region: standard deviation*.*


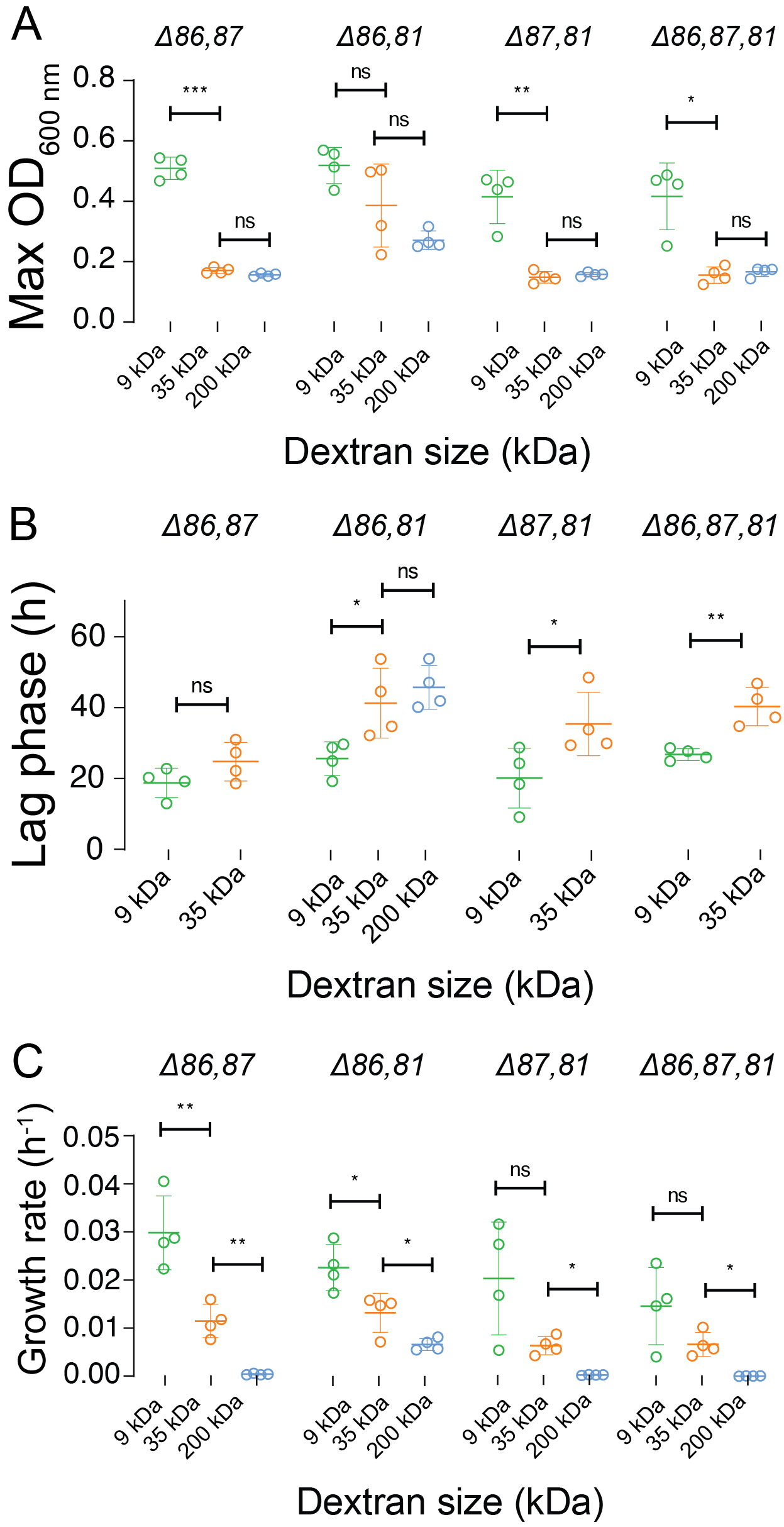


**Figure S3: Growth of *Bt* double and triple GH mutants in 3 different sizes of dextran.** (A) Quantified maximum OD of each mutant monocultures in dextrans (B) Quantified lag phase of double and triple GH mutants in 3 different sizes of dextrans. (C) Quantified growth rates of double and triple GH mutants in 3 different sizes of dextrans. All growth experiments: *N = 4,* error bars: standard deviation. Statistical test: *t-*tests were performed between pairs of dextran sizes*.* ns *p* > 0.05, * *p* < 0.05, ** 0.05 < *p* < 0.01, *** 0.001 < *p* < 0.0001.

**
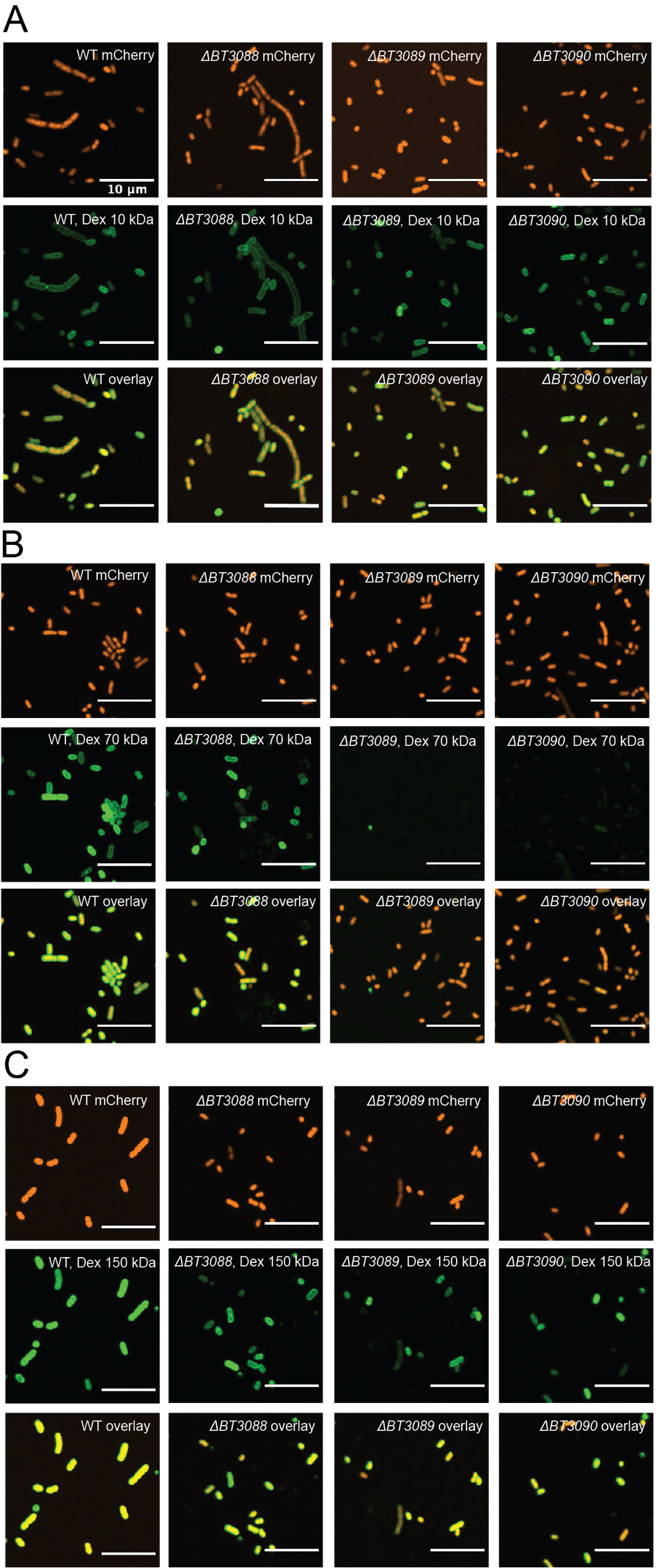
**

**Figure S4: Visualization of dextran localization to *Bt* cells.** Fluorescence microscopy images of *Bt* wildtype along with various mutants constitutively expressing mChery incubated in (A) dextran 10 kDa, (B) dextran 70 kDa, and (C) dextran 150 kDa. All dextrans conjugated to fluorescein.


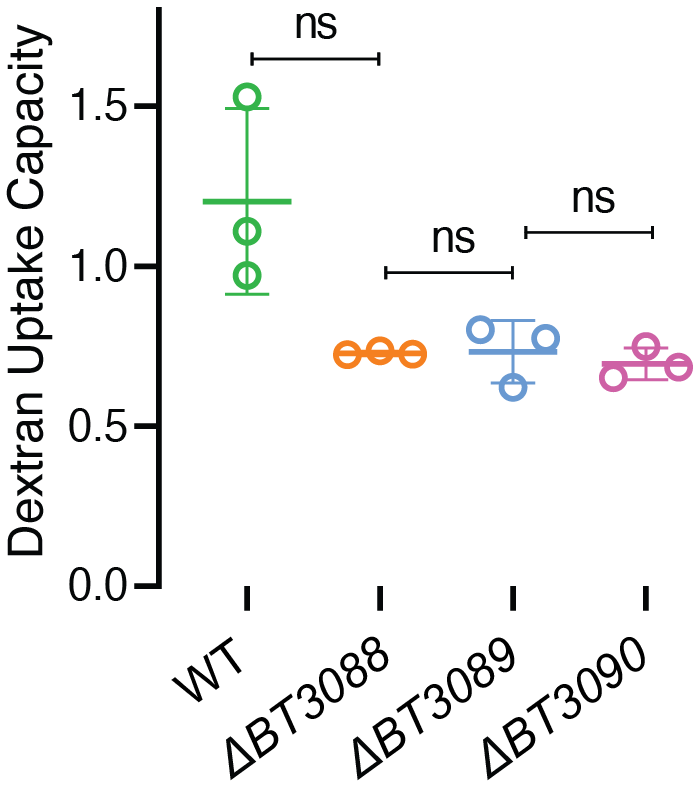


**Figure S5: Dextran uptake capacity of 10 kDa dextran by *Bt* WT and 3 PUL48 mutants.** No significant difference in the uptake capacity of dextran 10 kDa was observed across the 4 strains analyzed. *N = 3*, Statistical test: Statistical test: unpaired t-tests, ns *p* > 0.05.
